# Hierarchical feature alignment for cross-view geo-localization

**DOI:** 10.1038/s41598-026-48726-6

**Published:** 2026-04-24

**Authors:** Fuwen Su, Ziyang Zhou, Huajun Zhang, Huawei Zhang

**Affiliations:** 1 School of Automation, Wuhan University of Technology, Wuhan, 430070 China; 2 School of Information Engineering, Wuhan University of Technology, Wuhan, 430070 China; 3 School of Information Network Security, Xinjiang University of Political Science and Law, Tumushuke, 843900 China

**Keywords:** Cross-view geo-localization, Image retrieval, Hierarchical framework, Siamese network, Engineering, Mathematics and computing

## Abstract

Cross-view geo-localization aims to estimate the geographic coordinates of a street-view query by matching it with an aerial image database containing geotags. However, this task is fundamentally challenged by extreme viewpoint transformations and scale inconsistencies, which hinder the extraction of stable global structures and fine-grained local details. To address these limitations, we propose the GeoAlignNet (GANet), a hierarchical feature learning framework designed to achieve robust cross-scale and cross-view representation alignment for retrieval-based geo-localization. Specifically, GANet comprises two complementary components. First, the Spatial Structure Attention (SSA) module performs structure-aware aggregation by combining window-based attention with adaptive window partitioning and enhanced positional encoding, enabling the network to capture view-invariant spatial layouts and to alleviate spatial misalignment induced by strong perspective changes. Second, the Local Representation Refinement (LRP) module adopts depthwise separable convolutions and a multi-scale gating mechanism to optimally model fine-grained local feature representations, so as to improve the perception of geometric textures and achieve stable characterization against appearance variations and environmental noise. To further improve retrieval discriminability, we adopt a hybrid objective that jointly enforces intra-class compactness and inter-class separability in the embedding space, facilitating stable optimization under extreme cross-domain structural variations. Extensive experiments demonstrate that GANet achieves competitive performance compared with existing cross-view geo-localization methods, highlighting its strong effectiveness and generalization capability.

## Introduction

Image-based geo-localization aims to determine the location of a query street-view image by retrieving the most visually similar image from a GPS-tagged satellite imagery database. In cross-view localization, queries come from street-view images, while the reference database consists of satellite or aerial imagery. This capability supports applications such as autonomous driving^1^, robotic navigation^2^ and augmented reality^8^. A key difficulty is that the two views differ in both appearance and geometry. Changes in viewpoint reshape the spatial layout, so the same place may share similar semantics but still be hard to align structurally. Scale mismatch further widens the gap. A local texture in a street-view image can correspond to a much larger area in satellite imagery, while details may be weak from the ground or not visible at all. As a result, semantic correspondences become unstable and retrieval accuracy drops. Occlusions and moving objects make this issue more severe. Cross-view matching should therefore model regional structural changes, keep spatial relationships and align semantics across views.

**Fig. 1:**
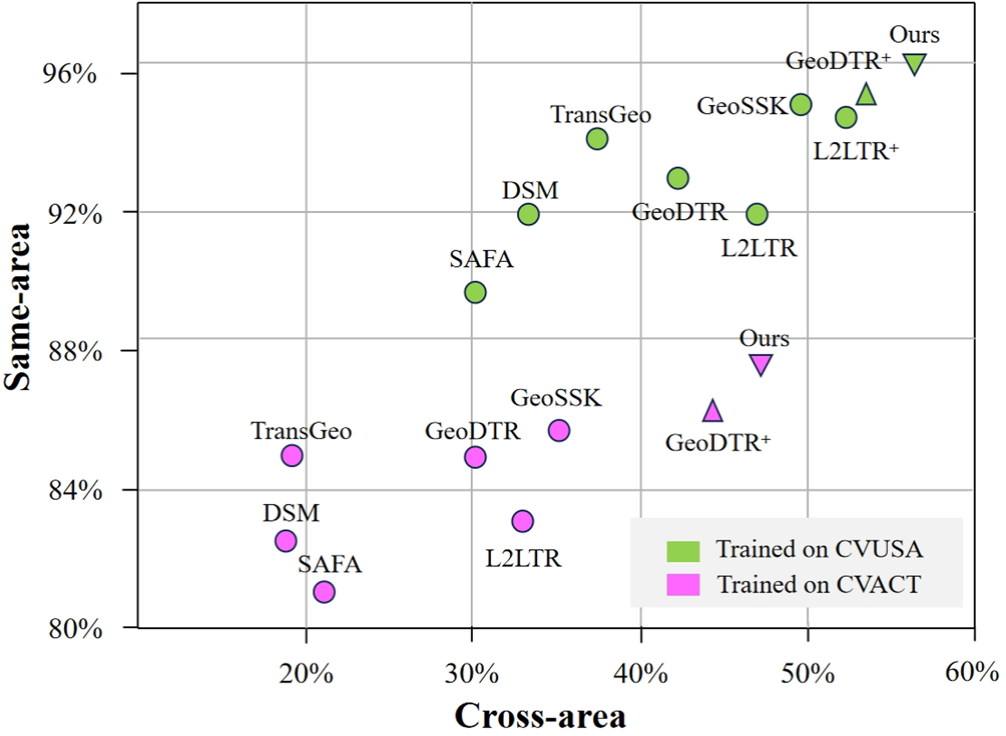
Compare the R@1 accuracy in same-area and cross-area. $\bigtriangleup$ denotes the baseline (GeoDTR), while $\bigtriangledown$ represents our method. The experimental results demonstrate that our method exhibits strong competitiveness in both scenarios.

Existing geo-location methods can be roughly categorized into two groups: feature-based approaches (e.g^4–7^.,) and geometry-based approaches (e.g^9–13^.,). Feature-based methods learn visual representations and perform matching in an embedding space, usually by optimizing feature similarity between street-view and aerial images. Geometry-based methods exploit spatial layout information, such as orientation, structure, or geometric consistency, to reduce cross-view ambiguity and improve alignment. In addition to these two major directions, recent work has also explored generative modeling as a complementary strategy for reducing the cross-view gap. For example, Arrabi et al.^14^ proposed a diffusion-based framework that synthesizes aerial images from ground views with geometry and text guidance, offering an alternative way to alleviate cross-view discrepancies. Those proposed by^4,5,9,10^, aim to address this challenge by employing convolutional neural networks (CNNs) for feature extraction to match street-view images with corresponding aerial images. For instance^5^, introduced a lightweight attention module to enhance model performance by emphasizing salient features within images. However, the traditional CNNs used in these methods are built on local receptive fields, so the learned representations are dominated by local textures and fragments. Global information are not integrated consistently^15^. demonstrate that spatial resolution critically influences feature representation and semantic reliability across scales, underscoring the importance of multi-scale modeling and spatially aware feature extraction for robust cross-view geo-localization. To address this^16^, introduced an transformer-based model that learns features from a broader spatial scope and uses them for cross-view retrieval. However, these methods do not sufficiently incorporate geometric layout information during feature extraction while scale inconsistency further increases cross-view misalignment, leading to unstable retrieval.

Despite recent progress, existing cross-view geo-localization methods primarily optimize for intra-region performance, often failing to maintain accuracy in cross-area scenarios where domain shifts are more pronounced. From a transfer learning perspective, remote sensing applications often require models trained in one region, season, or sensor configuration to remain effective when deployed elsewhere under domain shifts. Similar cross-domain transfer demands have been observed in diverse satellite-based tasks such as forest cover classification, peatland carbon-emission assessment, long-term lake/reservoir water storage monitoring, and cyanobacterial bloom magnitude estimation^17–20^. These observations further motivate cross-view geo-localization methods to emphasize cross-area transferability, rather than optimizing only for intra-region retrieval. As highlighted in^21^ and further quantified by semantic-graph investigations in^22^, this performance degradation stems from two critical deficiencies: the neglect of explicit geometric spatial alignment and inadequate extraction of global contextual semantics. Traditional Convolutional Neural Network (CNN) architectures, while effective at capturing local textures, rely on localized receptive fields that cannot maintain stable correspondences under the drastic viewpoint and scale transformations inherent in street-to-aerial matching. Although recent Transformer-based models have emerged to address long-range dependencies, they frequently overlook the intricate geometric layout information and lack the multi-scale adaptability required for complex, high-resolution geographic scenes.

Inspired by the Swin Transformer^23^, we build the GANet framework to address two central challenges in cross-view scenarios, significant scale variations and semantic misalignment. To address the structural and semantic discrepancies between cross-view domains, we persent the Spatial Structure Attention (SSA) module. Cross-view images often exhibit inconsistent geometric layouts and uneven spatial detail distribution, making fixed attention windows insufficient for reliable feature extraction. SSA therefore adjusts the attention scope according to regional structure. Specifically, it estimates local density and spatial layout to determine an appropriate window size for each region. In regions containing rich fine-grained details, the window is reduced so that the model can focus on discriminative local patterns. In contrast, in spatially sparse or open regions, the window is enlarged to capture broader contextual information. In addition, SSA incorporates relative spatial relationships into the attention process, which helps preserve geometric consistency across scenes and geographic areas. Through this adaptive design, SSA improves structural alignment and supports more robust feature learning in cross-view matching. Drawing inspiration from ConvNeXt24^24^, we develop the Local Representation Refinement (LRP) module to improve feature robustness under cross-view geometric variations. Cross-view matching often suffers from viewpoint-induced distortions and large scale changes, which can weaken the stability of local representations. To address this issue, LRP adopts a multi-scale refinement strategy that integrates fine-grained local patterns with broader regional context. Specifically, the module enhances local detail modeling while preserving structural cues at a larger spatial range. This design allows the network to produce more stable features when geometric appearance changes substantially across views. As a result, LRP improves the model’s robustness to geometric distortions and strengthens generalization across different geographic areas.

Moreover, we implement a Hybrid Loss strategy to optimize the cross-view embedding space against severe domain noise and perspective shifts. While Triplet loss establishes the foundational metric structure, its reliance on isolated sample pairs is insufficient for modeling complex cross-domain distributions. We overcome this by integrating Supervised Contrastive (SupCon) loss^25^ to enforce global batch-level cohesion through multi-sample clusters, and Circle loss^26^ to adaptively re-weight pairwise similarities. This hybrid approach strengthens intra-class compactness and sharpens decision boundaries, ensuring a discriminative and robust representation. The performance of GANet on standard cross-view geo-localization benchmarks is shown in Fig. 1. Here, the $\bigtriangleup$ denote the baseline, $\circ$ denote other methods and the $\bigtriangledown$ represent our proposed method.

Our main contributions can be summarized as follows:

- In the Geo-Feature Encoder, SSA module is to tackle viewpoint-induced misalignment ensuring that geometric relationships remain invariant even under significant scale shifts. LRP is developed to preserve critical fine-grained details that are prone to degradation in cross-view matching, enabling more stable and discriminative local representations.
- The Hybrid Loss strategy is impelemted to optimize the cross-view embedding space. Specifically, SupCon loss is employed to enforce global batch-level consistency, while Circle Loss adaptively emphasizes informative pairwise relationships, jointly improving embedding compactness and discriminative separability.
- Extensive experiments demonstrate that the porposed GANet exhibits strong competitiveness in both intra-region and cross-region geolocation tasks.

**Fig. 2:**
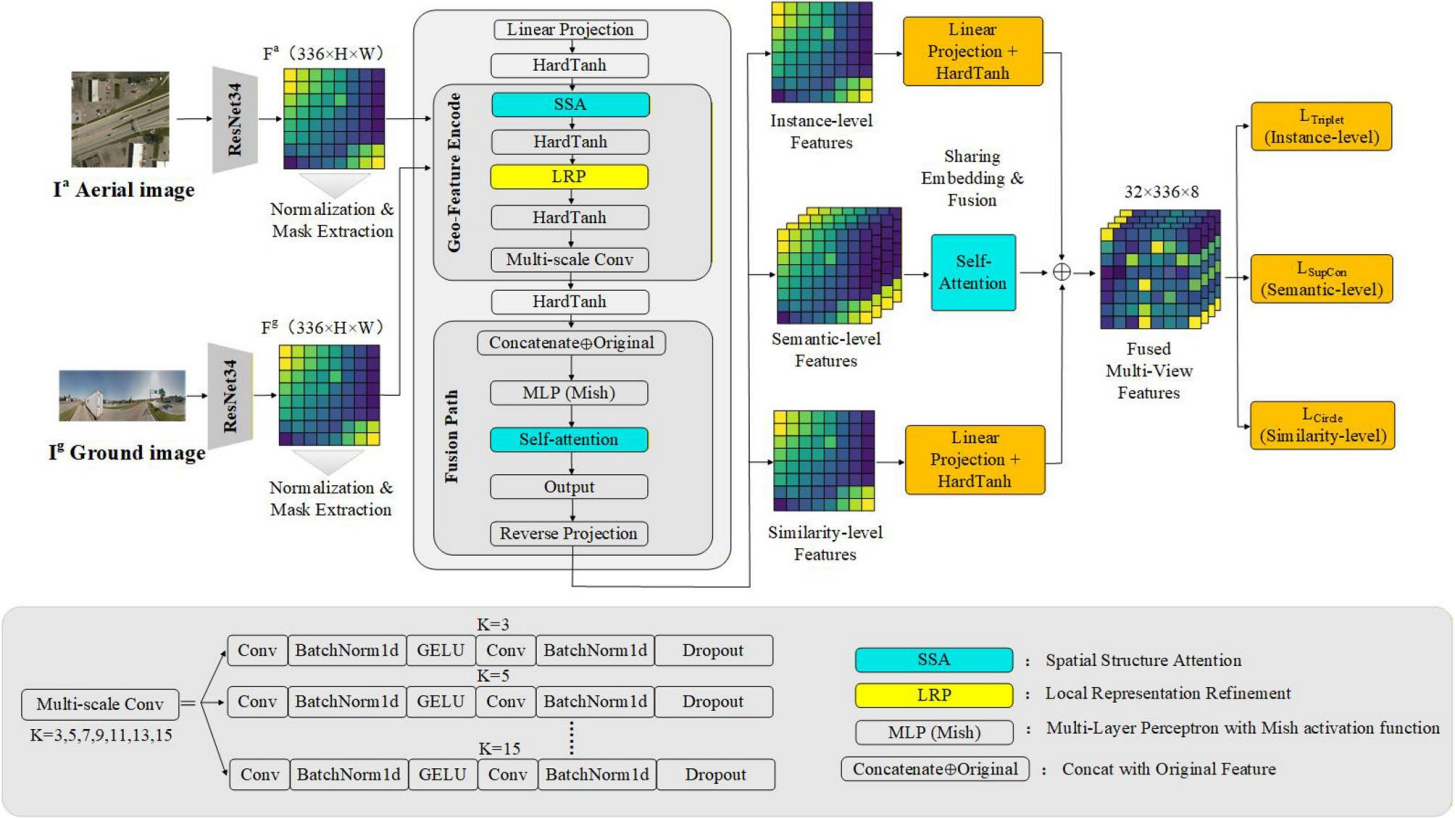
Overview of our proposed Model. The network applies a Siamese-like architecture (no weight-shared) for extracting features from the two views. First, CNN capture the low-level features of each input. Then, the features are refined by normalization and mask extraction, and fed into the proposed Geo-Feature Encoder, which incorporates SSA and LRP to Encoder geometry-aware representations. After projection and normalization, multi-view features are fused into a shared embedding. Finally, we impose supervision at different representation levels and get the correct match. The bottom part of the figure serves as a legend, explaining the module symbols.

## Related work

Cross-view geo-localization has attracted extensive attention in recent years. A broader overview of image and object geo-localization can be found in the survey by Wilson et al.^3^. In the following, we review the most relevant literature from the perspectives of problem formulation, feature-based methods and geometry-based methods.

###  Problem formulation

Statement of the Problem. The objective of cross-view geo-localization is to retrieve the ground-level image that best matches a given aerial image with known location information. Specifically, we assume there exists a set of ground-view images $\{g_i\}_{i=1}^{M}$ and a set of aerial images $\{a_j\}_{j=1}^{N}$, where *g* and *a* denote ground and aerial images respectively, and *M* and *N* are the total numbers of images in each domain. Given a query ground image q, the goal is to identify the corresponding aerial image by learning discriminative latent representations. The essence of the task is to use the Euclidean distance to measure the similarity between two images, thereby bringing matched image pairs closer to each other and pushing unmatched image pairs farther apart in the embedding space. The goal is to quantify the distance to find the minimum value, and then obtain the most matched aerial-view image, as shown in the following formula:

1 $$\begin{aligned} b = \mathop {\textrm{argmin}}_{i \in \{1, \ldots , N\}} d(f_q^g, f_i^a) \end{aligned}$$

where $d(\cdot , \cdot )$ the Euclidean distance, and *q* is the index of the query image. $f^g_q$ and $f^a_i$ represent the ground-view image and the aerial-view image respectively. *b* is the image index corresponding to the minimum distance.

###  Feature-based cross-view geo-localization

Feature-based localization tasks typically adopt CNN frameworks that focus on learning discriminative image representations^8^. first introduce CNNs to cross-view matching, drawing inspiration from the success of CNNs in the computer vision^27^. In their work, they also presented CVUSA which is nowadays one of the main benchmarks for cross-view geolocalisation^6^. and^4^employed dual-stream CNN architectures trained with Loss functions for view matching^5^. and^7^ further explored this direction by incorporating a lightweight attention module and leveraging triplet Loss (HER) and SEH, respectively, to learn geometric or semantic relationships between different views. In addition, prior studies have investigated various feature aggregation strategies and feature transformation techniques. For example, feature fusion^28^employs conditional GANs to synthesize aerial views from ground panoramic images as supervision, jointly extracts and adaptively fuses multi-scale features of both original and synthesized images, and realizes end-to-end cross-view matching to enhance cross-view representation learning^29^. incorporate the NetVlad architecture^30^ into a dual-branch VGG backbone^31^, aiming to derive viewpoint-invariant feature representations. Additionally, they introduce a weighted soft margin ranking loss to accelerate the network training process. While these methods learn cross-view representations, cross-view settings introduce distribution shift and often reduce available supervision, which makes feature matching less stable^32^. aligns source and target distributions via an optimal transport objective and imposes a low-rank constraint to reduce the influence of noisy source samples during alignment^33^. learns a visual–semantic projection, models semantic structure with a low-rank grouping prior, and uses graph regularization to propagate supervision to unlabeled data. But they do not directly resolve the structural mismatch in cross-view retrieval.

Even with distribution alignment and label propagation, cross-view retrieval still faces viewpoint-induced structural mismatch and scale inconsistency. Recent studies also show that multi hierarchy feature learning can improve matching robustness when cross-view evidence is ambiguous at a single granularity. Specifically, U-Match^34^ investigates multi hierarchy feature learning for two-view correspondence learning by aggregating local context across multiple hierarchies, which helps stabilize matching under large appearance changes. RefAtomNet++^35^ further demonstrates the effectiveness of multi hierarchy feature learning through semantic retrieval based aggregation, improving representation robustness under complex variations. Although these methods target correspondence estimation or video understanding rather than cross-view geo-localization retrieval, they provide supportive evidence that multi hierarchy feature learning is beneficial under severe cross-domain ambiguity. As a result, many feature-based pipelines lack sufficient global modeling and show limited scale adaptability, which leads to suboptimal retrieval performance. In this study, we address these limitations by designing the SSA and the LRP modules, leveraging the former’s capabilities in global modeling and geometric relation learning, and the latter’s strengths in local detail extraction and multi-scale feature capture. This combination compensates for existing shortcomings in feature alignment and scale adaptability.

###  Geometry-based cross-view geo-localization

Geometry-based methods aim to establish spatial correspondences between ground-level and aerial images, thereby avoiding ambiguities caused by geometric mismatches. This approach has recently emerged as a prominent research direction^9^. Encoderd pixel-level information by embedding angular orientation into the network for each pixel, enabling the model to learn spatial relationships^10^. proposed SAFA, which applies a Polar Transformation (PT) to aerial images to achieve geometric alignment with ground-view images. However, such methods are not well-suited for non-centrally aligned datasets, such as VIGOR^36^. Subsequently, the same authors proposed the Dynamic Similarity Matching (DSM) module^12^, this module estimates the azimuth angle using feature correlation to achieve the joint estimation of position and direction for restricted Field of View (FoV)^28^. adopted conditional generative adversarial networks (GANs)^11^ to learn cross-view geolocalization, complementary features of synthetic street images and aerial images are fused through joint feature learning, and multi-scale feature aggregation is employed to effectively reduce cross-view differences. Although previous studies have achieved notable progress, the inherent limitations of CNNs restrict the model’s ability to capture only local information. GeoDTR^13^ learns global information by extracting geometric descriptors while also incorporating data augmentation and counterfactual learning. However, this method suffers from insufficient feature utilization and poor dimensional adaptability. To address the above issues, we propose the GANet, which effectively leverages multi-scale information to reduce the differences between different viewpoints. Our model captures global spatial dependencies through hierarchical attention and uses enhanced convolutions to extract fine-grained local features. This approach enables more efficient modeling of the complex correspondences between aerial and ground-level viewpoints.

Recently, an increasing number of methods have focused on capturing global dependencies within images. For example, TransGeo^16^ abandons the traditional CNNs architecture and is the first to adopt a pure Transformer-based approach for learning image correspondences. MGTL^37^ addresses cross-view geolocalization by cascading attention masks within a Transformer framework. In addition, L2LTR^38^ introduces an innovative layer-to-layer Transformer architecture, further advancing global feature modeling. The aforementioned methods primarily improve cross-view matching by strengthening global dependency modeling or introducing implicit spatial reasoning. However, preserving spatial structure consistency under large viewpoint and scale variations remains challenging. Our method addresses this issue by explicitly modeling structural correspondence and reducing feature misalignment through geometry-aware representation learning, while avoiding the two-stage training strategy required by TransGeo^16^.

Building upon these advances, recent studies have extended geometry-aware cross-view geo-localization toward more realistic settings. Ye et al.^39^ proposed a Panorama-BEV Co-Retrieval Network that explicitly transforms street-view panoramas into BEV representations to narrow the perspective gap between ground and satellite imagery. Their method combines a panorama branch for global layout perception with a BEV branch for local detail matching and is further evaluated on the CVGlobal dataset under cross-regional, cross-temporal and street-to-satellite settings. Xia et al.^40^ investigated the decentrality problem, in which the query street-view image is spatially offset from the center of the aerial reference image. They introduced the DReSS dataset and the AuxGeo framework, where a BEV intermediary module and a position constraint module are used to improve robustness to spatially offsets. These works highlight that cross-view geo-localization increasingly requires both effective geometric modeling and evaluation under more realistic conditions. Our method differs in focus from these studies, instead of using explicit BEV co-retrieval or auxiliary position-prior supervision, we concentrate on adaptive geometry-aware feature learning and local representation refinement to improve robustness in both intra-region and cross-area retrieval.

**Fig. 3:**
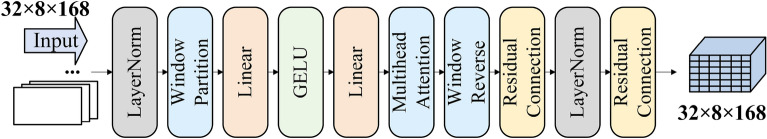
Illustration of the SSA framework.

**Fig. 4:**
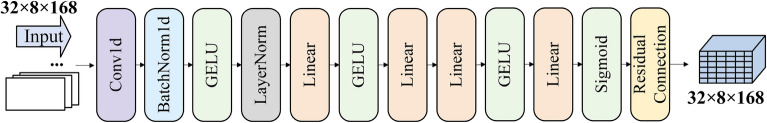
Illustration of the LRP framework.

## Methodology

###  GANet model

Overview of Model. This model adopts a Siamese-like architecture for the ground and aerial views, as illustrated in Fig. 2. In the backbone pathway, the two streams are processed in parallel. Given an input image $I^{v}$ ($v \in \{a,g\}$, the same convention applies hereinafter), the ResNet34 first extracts a feature map $\textbf{F}^{v}$. The features are then refined by position normalization and mask extraction to align spatial layouts and suppress background responses.

Next, the refined features are fed into the Geo-Feature Encoder, which integrates the proposed SSA and LRP modules to Encoder geometry-aware cues and produce a geometric descriptor. SSA targets viewpoint-induced misalignment by modeling geometry relations that remain stable under scale changes, while LRP preserves fine-grained structural details via depthwise separable convolutions and adaptive gating. The resulting descriptor modulates $\textbf{F}^{v}$ and is further projected through linear layers with HardTanh normalization to obtain the final representation.

Training uses a unified embedding and applies supervision at three complementary levels. The triplet loss provides the instance-level constraint by enforcing relative distance ordering among matched and mismatched pairs. The SupCon loss imposes a semantic-level constraint, pulling samples with the same label into compact clusters to maintain cross-domain semantic consistency. The circle loss further calibrates the similarity distribution by re-weighting positive and negative pairs, which stabilizes the ranking under hard samples without changing the matching definition.

### Geo-feature encoder

The Geo-Feature Encoder extracts high-level spatial structure information from visual features, aiming to construct a geometric layout representation with view invariance. In cross-view geolocalization tasks, image content is prone to changes due to external factors such as weather and lighting. However, the visual arrangement of scenes usually remains stable. Therefore, this paper enhances the discriminative ability and generalization ability of feature representation by modeling the geometric relationships between different views.

As shown in Fig. 2, the Geo-Feature Encoder begins with a preprocessing stage that includes position normalization and mask extraction. Position normalization aligns the spatial distributions across different viewpoints, while the mask extraction module highlights geometrically discriminative regions and suppresses background noise. The resulting normalized and masked features are used as the input for subsequent geometric encoding. The preprocessed features are then fed into the SSA module. Based on sliding-window multi-head self-attention, SSA performs cross-region structural alignment and feature reorganization through adaptive window configuration. This process produces an embedding vector *e* and outputs a feature tensor of size $32 \times 8 \times 168$. After structural alignment, the features are passed to the LRP module. By incorporating contextual information, LRP enhances local geometric patterns on the aligned features, allowing the representation to maintain structural stability while improving local consistency. The output is subsequently forwarded to multi-scale convolution modules to support spatial structure modeling at different granularities. At this point, the Fusion Path comes into play. The features from the aerial and ground images are concatenated, combining both sources of information. A MLP, with a Mish activation function, then refines the fused features. The fusion process is further enhanced by the Self-attention mechanism, which enables the model to selectively emphasize key regions while suppressing irrelevant parts across both views. The refined features are then processed through a Linear Projection to map them into a shared embedding space. Finally, the geometric layout descriptor $q \in \mathbb {R}^{32 \times 336 \times 8}$ is obtained. This descriptor captures the fused multi-view features and Encoders the spatial and semantic information required for accurate cross-view geo-localization.

**SSA.** The principle of the SSA is to replace the standard Multi-Head Attention (MSA) module with a 1D sequence window module equipped with an adaptive padding mechanism. As shown in Fig. 3, this module takes a 1D sequence as input and first undergoes Layer Normalization (LN) and window partitioning operations. Subsequently, the sequence position features are enhanced through a position enhancement module based on a MLP, and the processed features are input into the MSA module for attention calculation. After that, the sequence structure is restored through a window merging operation, and the original features are fused via residual connections. Finally, after processing by LN and MLP modules, a feature vector with the same shape as the input sequence is output.

**LRP.** To capture the multi-scale texture and structural features of geographic scenes, the LRP is constructed as shown in Fig. 4. This module takes a 1D sequence as input and first completes the initial feature transformation through depthwise separable convolution (Conv1d), BatchNorm1d, and the GELU activation function^41^. Then, after a LN, it sequentially uses pointwise convolution to expand and compress the feature dimensions. Subsequently, a feature refinement gating module composed of Linear layers, GELU, Linear layers, and Sigmoid is introduced to perform adaptive weighted refinement on the features. Finally, through residual connections, the final sequence features with the same shape as the input are output. LRP captures multi-scale texture and structural features of geographic scenes, and combines adaptive residual connections and feature refinement gating mechanisms. It enhances feature robustness while efficiently processing high-resolution geographic features, effectively alleviating cross-view visual differences and environmental noise interference.

###  Hybrid loss

To achieve cross-view geolocalization, we adopt a weighted Triplet Loss^42^ as the basic metric learning objective firstly:

2 $$\begin{aligned} L_{\text {triplet}} = \log \left( 1 + e^{\alpha (d_{\text {pos}} - d_{\text {neg}})}\right) \end{aligned}$$

where $d_{\text {pos}}$ and $d_{\text {neg}}$ denote the L2 distances between the positive and negative sample pairs in the same embedding space, respectively. The parameter $\alpha$ is a hyperparameter used to control the convergence rate, and its value is empirically determined.

Triplet Loss provides basic metric constraint for cross-view retrieval, requiring the anchor to be closer to the positive sample and farther from the negative sample. However, cross-view geolocalization involves severe domain noise and structural variations. Viewpoint and scale inconsistency between street-view and satellite imagery makes correspondences unstable. As a result, many easy negatives are far away and offer little leverage to further separate hard negatives. Moreover, Triplet uses only one positive and one negative per update and overlooks batch-level relationships, so intra-class cohesion and inter-class separation are hard to keep stable under different views. To maintain a compact and discriminative embedding space, we introduce SupCon Loss, which performs contrastive learning with multiple positives and many negatives within a mini-batch, leading to more intra-class cohesion and inter-class separation. We then incorporate Circle Loss, which adaptively reweights similarity scores and emphasizes sample relations that remain insufficiently optimized under scale inconsistency and cross-view misalignment. Triplet establishes the basic ranking, SupCon strengthens clustering and margins, and Circle focuses on hard relations to drive further separation, resulting in a compact and separable embedding space, which are defined as follows:

3 $$\begin{aligned} {L}_{\text {SupCon}} = - \frac{1}{N} \sum _{i=1}^{N} \frac{1}{|P_i|} \sum _{j \in P_i} \log \left( \frac{ \exp \left( \textbf{z}_i \cdot \textbf{z}_j / \tau \right) }{ \sum _{k \notin N_i} \exp \left( \textbf{z}_i \cdot \textbf{z}_k / \tau \right) } \right) \end{aligned}$$

where $P_i$ denotes the set of all positive samples for anchor sample *i*, $N_i$ denotes the set of all negative samples and the $\tau$ is temperature. $\textbf{z}_i \cdot \textbf{z}_j$ and $\textbf{z}_i \cdot \textbf{z}_k$ measure the similarity between two feature vectors.

4 $$\begin{aligned} {L}_{\text {Circle}} = \log \left( 1 + \sum _{\text {pos}} e^{\gamma a_{\text {pos}} (s_{\text {pos}} - \Delta _{\text {pos}})} + \sum _{\text {neg}} e^{-\gamma a_{\text {neg}} (s_{\text {neg}} - \Delta _{\text {neg}})} \right) \end{aligned}$$

Where, $a_{\text {pos}}$ and $a_{\text {neg}}$ are the activation functions for positive samples and negative samples, respectively. $\Delta _{\text {pos}} = 1 - m$ and $\Delta _{\text {neg}} = m$ (*m* is set to 0.25). The $\gamma$ controls the optimization intensity. The final Loss can be expressed as:

5 $$\begin{aligned} L = L_{\text {triplet}} + \alpha \cdot L_{\text {supcon}} + \beta \cdot L_{\text {circle}} \end{aligned}$$

where $$\alpha = 0.2$$ and $$\beta = 0.05$$.

In summary, this Loss function takes into account the balance of sample differentiation difficulty and constrains the feature distance between positive and negative samples. Its purpose is to address the problems that the model tends to fall into local optima and the global sample relationships are overlooked.

**Table 1:** Comparisons between GANet (Ours) and state-of-the-art methods on the CVUSA dataset. $\dagger$ indicates applying PT to aerial images. Best and second-best results are shown in bold and underlined, respectively. We also report computation overhead including GFLOPs, number of parameters and inference time, to facilitate efficiency comparisons.

Method	R@1	R@5	R@10	R@1%	GFLOPs	Params	Inference Time
FusionGAN^43^	48.75%	–	81.27%	95.98%	-	-	-
CVFT^44^	61.43%	84.69%	90.49%	99.02%	28.5	26.8 M	38 ms
SAFA†^10^	89.84%	96.93%	98.14%	99.64%	42.2	67.1 M	40 ms
DSM†^12^	91.93%	97.50%	98.54%	99.67%	39.3	14.5 M	-
CDE†^45^	92.56%	97.55%	98.33%	99.57%	64.9	142.7 M	139 ms
L2LTR†^38^	94.05%	98.27%	98.99%	99.67%	46.8	195.9 M	146 ms
TransGeo^16^	94.08%	98.36%	99.04%	99.74%	11.4	44.2 M	36 ms
TransGCNN^46^	94.15%	98.21%	98.94%	99.79%	-	-	-
SEH†^7^	95.11%	98.45%	99.00%	99.78%	-	-	-
Li et al.^47^	93.53%	98.42%	99.18%	99.77%	-	-	-
GeoDTR^13^	93.76%	98.47%	99.22%	99.85%	40.2	48.5 M	85 ms
GeoDTR†^13^	95.43%	98.86%	99.34%	**99.86%**	40.5	49.1 M	87 ms
MB-JRLN-IFS†^48^	95.09%	98.85%	99.34%	99.77%	-	-	-
GeoSSK^49^	94.13%	98.75%	99.27%	99.85%	-	-	-
OriLoc^50^	95.97%	98.42%	99.23%	99.56%	-	-	-
TriViTs^51^	95.35%	98.82%	99.29%	99.80%	-	-	-
GeoDTR+^58^	95.40%	98.44%	99.05%	99.75%	11.3	24.7 M	38 ms
**GANet**	94.74%	98.82%	99.38%	99.85%	45.8	109.7 M	112 ms
**GANet†**	**96.38%**	**99.12%**	**99.41%**	99.85%	46.2	113.8 M	118 ms

**Table 2:** Comparisons between GANet (Ours) and state-of-the-art methods on the CVACT_val and CVACT_test datasets. Notations are the same as Table I.

Method	CVACT_val				CVACT_test			
	R@1	R@5	R@10	R@1%	R@1	R@5	R@10	R@1%
CVFT^44^	61.05%	81.33%	86.52%	95.93%	26.12%	45.33%	53.80%	71.69%
SAFA†^10^	81.03%	92.80%	94.84%	98.17%	55.50%	79.94%	85.08%	94.49%
DSM†^12^	82.49%	92.44%	93.99%	97.32%	35.63%	60.07%	69.10%	84.75%
CDE†^45^	83.28%	93.57%	95.42%	98.22%	61.29%	85.13%	89.14%	98.32%
L2LTR†^38^	84.89%	94.59%	95.96%	98.37%	60.72%	85.85%	89.88%	96.12%
TransGeo^16^	84.95%	94.14%	95.78%	98.37%	-	-	-	-
TransGCNN^46^	84.92%	94.46%	95.88%	98.36%	-	-	-	-
SEH†^7^	84.75%	93.97%	95.46%	98.11%	-	-	-	-
Li et al.^47^	84.44%	94.85%	96.15%	98.53%	-	-	-	-
GeoDTR^13^	85.43%	94.81%	96.11%	98.26%	62.96%	87.35%	90.70%	98.61%
GeoDTR†^13^	86.21%	95.44%	96.72%	98.77%	64.52%	88.59%	91.96%	**98.74%**
MB-JRLN-IFS†^48^	86.64%	94.61%	95.94%	98.45%	-	-	-	-
GeoSSK^49^	85.56%	94.93%	96.17%	98.54%	-	-	-	-
OriLoc^50^	86.23%	94.66%	95.87%	98.30%	-	-	-	-
TriViTs^51^	86.35%	95.67%	**97.07%**	98.61%	-	-	-	-
GeoDTR+^58^	**87.61%**	95.48%	96.52%	98.34%	**67.57%**	89.84%	92.57%	98.54%
**GANet**	87.00%	95.40%	96.77%	98.59%	65.64%	88.48%	91.92%	98.51%
**GANet**†	87.52%	**96.00%**	97.05%	**98.80%**	67.23%	**90.17%**	**92.81%**	98.58%

## Experients

###  Datasets and metric

We evaluate the effectiveness of GANet on two public datasets: CVUSA^8^ and CVACT^9^. Both datasets support standard and fine-grained cross-view geolocalization tasks and adopt a one-to-one retrieval setting. The proposed method is compared with^7,10,12,13,16,38,43–46,48–53^.

- CVUSA integrates ground and satellite imagery collected from multiple areas across the United States. The ground images are extracted from Google Street View, featuring a resolution of 1232 $\times$ 224 pixels, whereas the satellite imagery is acquired from Microsoft Bing Maps, with a zoom level of 19 and a resolution of 750 $\times$ 750 pixels. This dataset consists of 35,532 image pairs for training purposes and 8,884 pairs for testing. It represents the inaugural dataset to be used in a cross-view matching study. The ground images have been adjusted to match the orientation of the satellite images, enabling their use as an extra feature.
- CVACT offers a dataset of ground and satellite images from Canberra. Its ground-based panoramic images are sourced from Google Street View and have a resolution of 1664 $\times$ 832 pixels. The satellite imagery is obtained from Google Maps and features a 1200 $\times$ 1200 pixel resolution. The dataset includes 35,532 image pairs for training and 8,884 pairs for evaluation (CVACT_val).

**Table 3:** Comparisons between GANet (Ours) and state-of-the-art methods on cross-area benchmarks. Notations are the same as Table I.

Method	CVUSA$\rightarrow$CVACT				CVACT$\rightarrow$CVUSA			
	R@1	R@5	R@10	R@1%	R@1	R@5	R@10	R@1%
SAFA†^10^	30.40%	52.93%	62.29%	85.82%	21.45%	36.55%	43.79%	69.83%
DSM†^12^	33.66%	52.17%	59.74%	79.67%	18.47%	34.46%	42.28%	69.01%
TransGeo^16^	37.81%	61.57%	69.86%	89.14%	18.99%	38.24%	46.91%	88.94%
L2LTR^38^	47.55%	70.58%	-	91.39%	33.00%	51.87%	60.63%	84.79%
L2LTR†^38^	52.58%	75.81%	77.39%	93.51%	-	-	-	-
GeoDTR^13^	43.72%	66.99%	74.61%	91.83%	29.85%	49.25%	57.11%	82.47%
GeoDTR†^13^	53.16%	75.62%	81.90%	93.80%	44.07%	64.66%	72.08%	90.09%
AMPLE^52^	46.72%	64.99%	71.07%	87.47%	32.61%	47.03%	53.21%	74.43%
GeoSSK^49^	49.37%	71.75%	78.70%	92.34%	35.52%	54.07%	63.61%	87.18%
SEMGEO^53^	51.47%	75.20%	80.90%	93.90%	38.12%	59.66%	68.39%	89.96%
GeoDTR+^58^	**61.17%**	**80.22%**	**85.45%**	**94.56%**	**53.89%**	**74.56%**	**81.10%**	**94.93%**
**GANet**	50.60%	73.04%	79.38%	93.46%	36.46%	55.44%	63.45%	86.05%
**GANet†**	55.95%	77.14%	82.80%	94.08%	47.56%	67.03%	74.64%	91.68%

Following previous works^9,10,12,29,38,45,54^, we evaluate our model using the R@K metric with $K = \{1, 5, 10, 1\%\}$, which denotes the probability that a correct match is included in the top-$K$ retrieved results. In this search, we evaluate the performance of all methods on R@1, R@5, R@10, and R@1% under both same-area and cross-area scenarios. The specific calculation formulas are as follows:

6 $$\begin{aligned} \text {R@}K = \frac{1}{|Q|} \sum _{i=1}^{|Q|} \mathbb {I}\left( \text {rank}(q_i, c_{i,\text {true}}) \le K \right) \end{aligned}$$

Where, $K \in \{1, 5, 10, 0.01 \times |C|\}$, *Q* denotes the set of ground images, and *C* denotes the set of satellite images; $q_i$ represents the *i*-th ground image, and $c_i$ represents the true matching satellite image corresponding to the query $q_i$. $\mathbb {I}(\cdot )$ is used to indicate whether a single query is successfully matched.

###  Implementation details

We employ a ResNet-34^55^ as the backbone for a fair comparison with other baselines. $\alpha$ and $\beta$ are set to 10 and 5, respectively. We train the model for 500 epochs using the AdamW^56^ optimizer with a batch size of 32 and a weight decay of 1 $\times$ 10$^{-4}$. The training and testing processes are conducted using the PyTorch^57^ platform and an NVIDIA RTX 4090 GPU.

###  Same-area experiment

We first evaluate our model on the same-area and cross-view geo-localization tasks. The results on the CVUSA, CVACT_val and CVACT_test datasets are shown in Table 1 and Table 2, respectively. We trained the model under two scenarios: one using PT and one without. Specifically, with PT, our model achieved competitive performance compared with existing methods.

Without PT, the GANet not only exceeds baseline (GeoDTR) but also outperforms GeoDTR†on R@10 in the CVUSA experiments. In the CVACT_val experiments, the GANet outperforms GeoDTR, achieving 87.00% on R@1 and 96.77% on R@10, which are 0.79% and 0.05% increases from GeoDTR†. To be noticed, the proposed method achieves comparable performance on R@1 of CVACT_test. This indicates that the fused structure of SSA and LRP can accurately capture regional features. The above experimental results demonstrate the superiority of the GANet.

Furthermore, we additionally report the computational overhead in Table 1, including GFLOPs, number of parameters and inference time. This makes the comparison more comprehensive. Compared with the baseline GeoDTR, GANet introduces richer feature enhancement modules, leading to moderately higher computational cost, with GFLOPs, parameters and inference time increasing from 40.2, 48.5M and 85 ms to 45.8, 109.7M and 112 ms, respectively. However, this additional overhead brings consistent gains in retrieval performance. More importantly, GANet still remains competitive in efficiency when compared with other methods such as CDE and L2LTR, which require substantially higher computational overhead but do not achieve better results on the CVUSA benchmark. Therefore, the proposed method achieves a favorable balance between localization accuracy and computational efficiency.

###  Cross-area experiment

To further demonstrate the model’s performance in object occlusion scenarios, we conducted cross-area training on CVUSA while testing on CVACT (CVUSA $\rightarrow$ CVACT) and vice versa. The experimental results are shown in Table 3. On the CVUSA$\rightarrow$CVACT task, the baseline GeoDTR achieved 53.16% in R@1, while GANet achieved 55.95%, representing a 2.79% improvement over GeoDTR. With PT, the accuracy reached 50.60%, showing a significant improvement over GeoDTR. More importantly, on the CVACT $\rightarrow$ CVUSA task, the R@1 accuracy of GeoDTR is 44.07% with PT and 29.85% without PT, while GANet improves the results to 47.56% (+3.49%) and 36.46% (+6.61%), respectively. The above results demonstrate that our proposed method possesses strong spatial generalization ability, while also exhibiting excellent robustness and practical value in cross-view area.

**Fig. 5:**
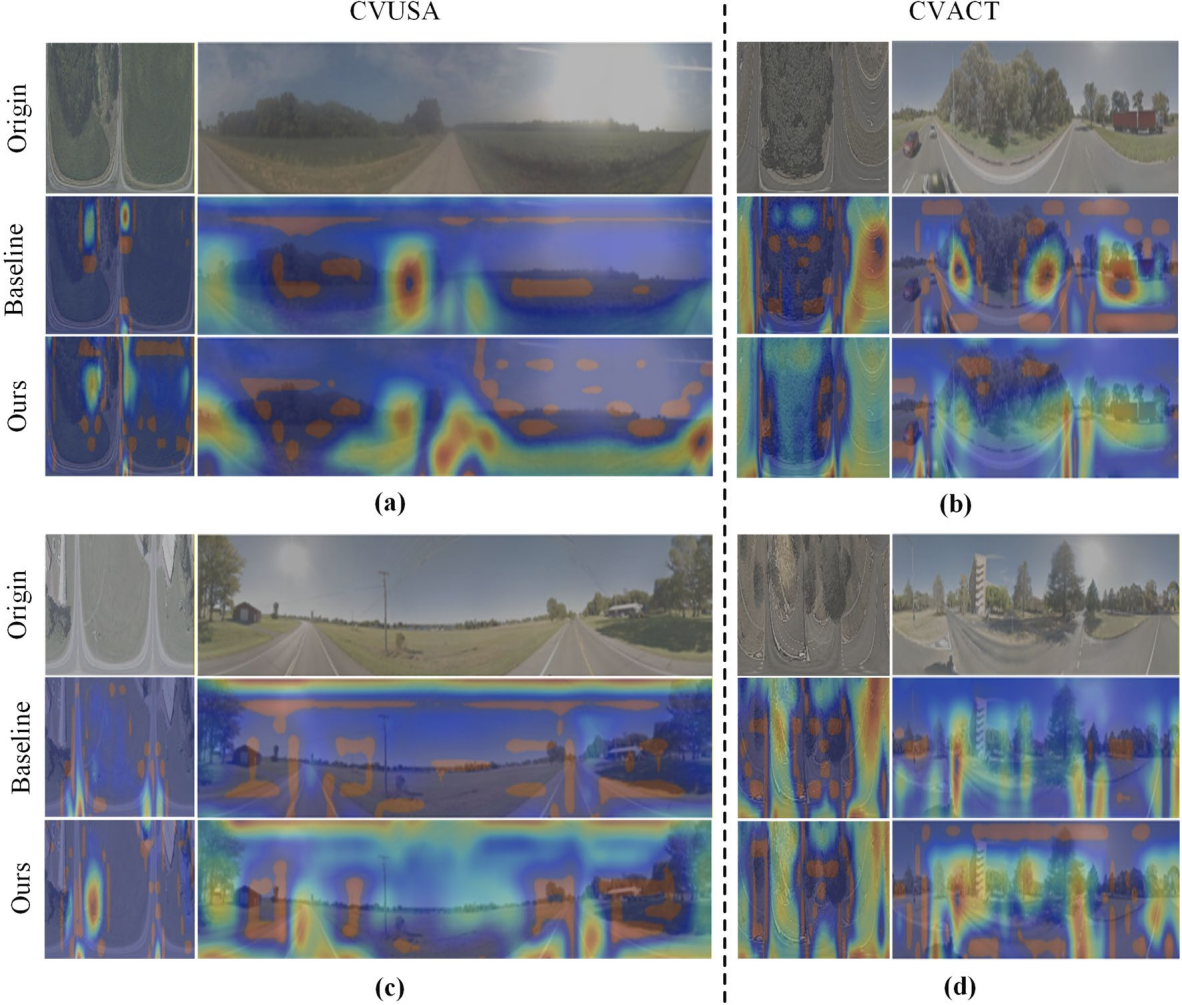
Heatmap visualization of the Baseline and Our model. On CVUSA and CVACT datasets, the original image, the baseline model’s heatmap, and the heatmap of our method are displayed from top to bottom. Each dataset provides two examples. It is discernible that the Baseline mainly focuses on road information, while Our model pays more attention to some salient buildings in addition to road information.

**Fig. 6:**
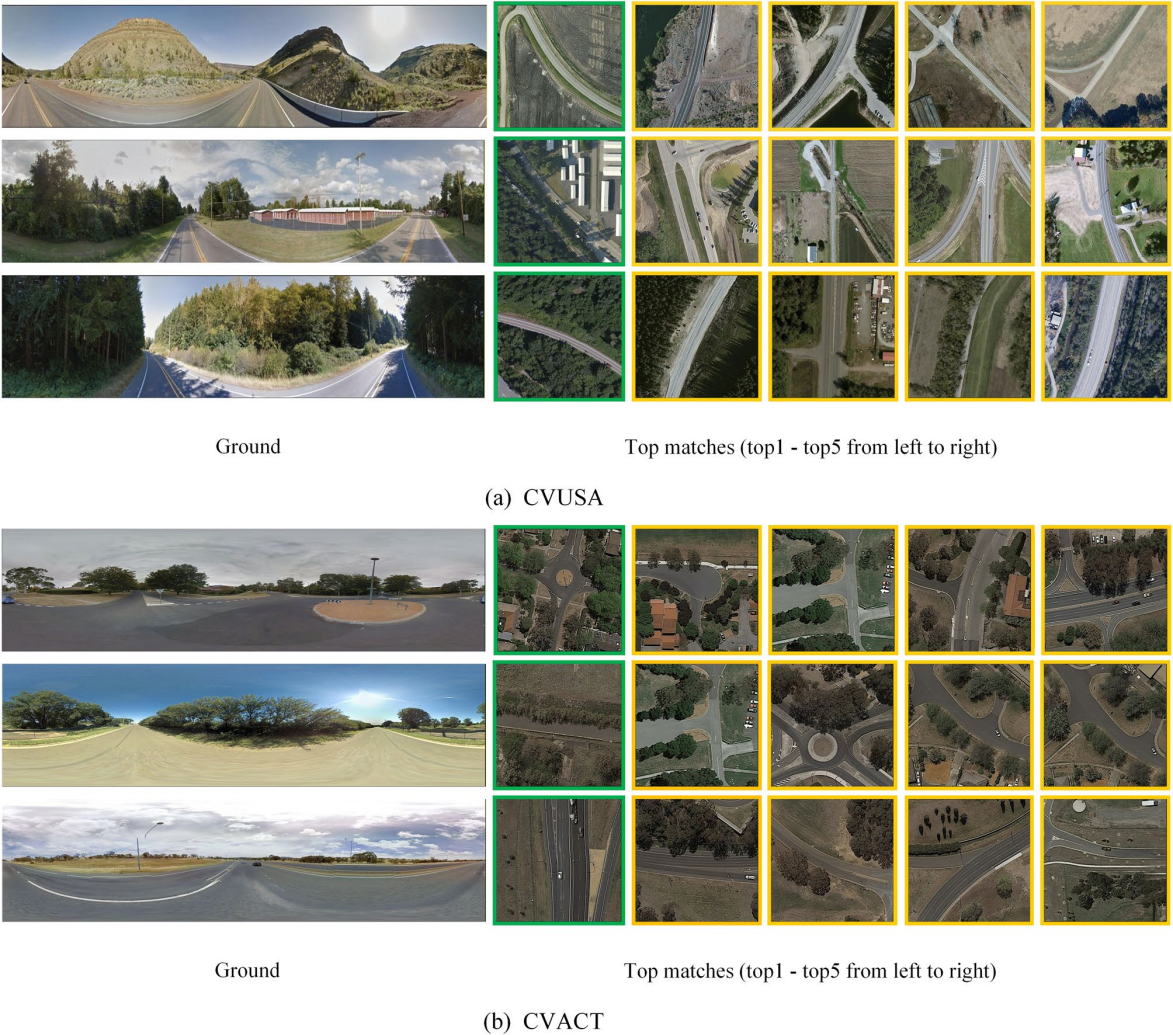
Top-5 recall image retrieval examples on (**a**) CVUSA dataset, (**b**) CVACT dataset. Each dataset provides three scene samples, and each row displays a query image followed by the five most similar reference images from left to right. Green and Yellow borders indicate correct and incorrect retrieved results, respectively.

**Fig. 7:**
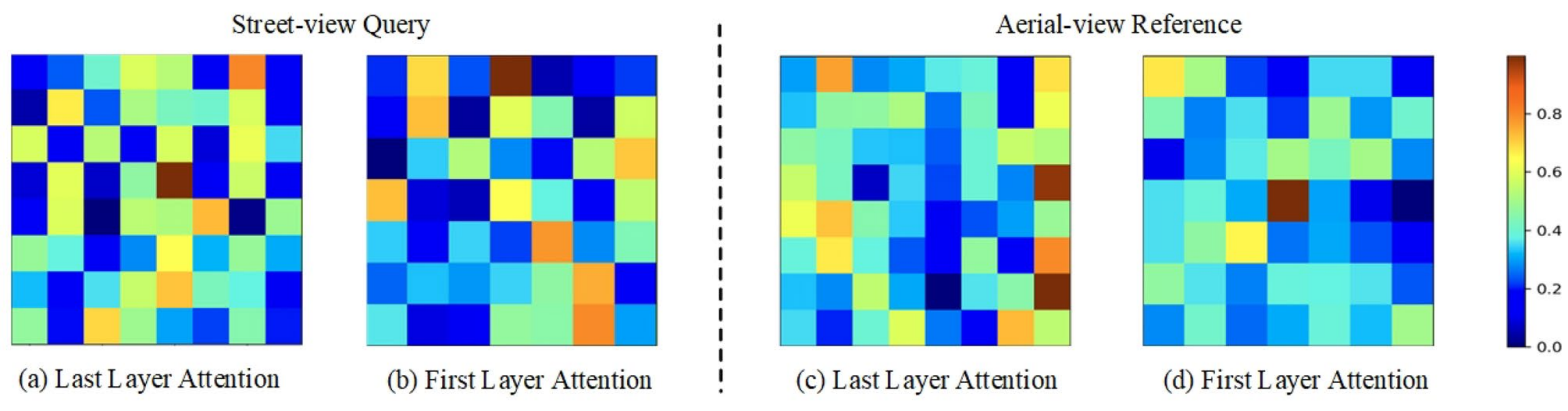
Visualization of attention maps extracted from the first and last layers of the Geo-Feature Encoder on CVUSA. The maps are computed on intermediate feature representations of paired Street-view queries and Aerial-view references. In the first layer, attention responses are relatively dispersed with multiple moderate-response regions, and the peak locations differ across views. In the last layer, attention becomes more selective, where fewer locations receive high responses and high-response sites stand out more clearly. This results in filtering view-specific noise and retaining geometry-relevant cues, which leads to more stable similarity estimation for cross-domain retrieval.

**Table 4:** Effectiveness of the proposed model under different configurations. Here, w/o denotes the removal of a specific module, and K represents the number of descriptors. The experiments are conducted on the CVUSA dataset.

Models	K	Loss	R@1	R@5	R@10	R@1%
Ours	8	Hybrid	96.38%	99.12%	99.41%	99.85%
Ours	8	Triplet	96.12%	99.04%	99.49%	99.83%
Ours	4	Triplet	95.78%	98.98%	98.32%	99.83%
Ours	2	Triplet	95.45%	98.78%	99.37%	99.81%
Ours w/o SSA	8	Hybrid	95.07%	98.64%	99.27%	99.83%
Ours w/o LRP	8	Hybrid	95.82%	99.00%	99.44%	99.81%
Ours w/o Geo-Feature Encoder	8	Hybrid	95.72%	99.00%	99.39%	99.80%

###  Ablation study

In our ablation study, we conducted an in-depth investigation into the effectiveness of the design choices made in this work, such as the model architecture or components.

**SSA.** As shown in Table 4, when the SSA module is removed, the model performance exhibits a systematic decline. Specifically, the accuracy drops by 1.31% on R@1, and R@10 shows a slight observable decrease. This result clearly confirms the module’s core role in the model architecture. The SSA module’s dynamic window size adjustment mechanism adaptively allocates modeling granularity based on the feature density of different regions. When combined with window-level attention mechanism, it weights and enhances key regional features, thereby effectively bridging the gap between global spatial layout information and local semantic details. Consequently, this ablation study not only shows the SSA module’s contribution to performance but also validates its irreplaceability as a link between global and local features. This dynamic modeling capability is particularly crucial for improving matching robustness in complex geographic scenarios.

**Table 5:** Effectiveness of the Hybrid Loss. The performance is reported on the CVUSA datasets without PT.

Method	CVUSA			
	R@1	R@5	R@10	R@1%
Baseline	92.49%	98.24%	98.90%	99.81%
Baseline+SupCon Loss	93.15%	98.50%	99.18%	99.85%
Baseline+CirCle Loss	92.96%	98.40%	99.14%	99.85%
Baseline+Hybrid Loss	93.81%	98.82%	99.36%	99.82%

**Table 6:** The selection of $\alpha$ and $\beta$.

Method	CVUSA			
	R@1	R@5	R@10	R@1%
$\alpha = 1.0$, $\beta = 0.5$	96.22%	99.09%	99.41%	99.84%
$\alpha = 0.8$, $\beta = 0.3$	96.12%	98.95%	99.38%	99.85%
$\alpha = 0.5$, $\beta = 0.15$	96.03%	99.03%	99.41%	99.84%
$\alpha = 0.3$, $\beta = 0.1$	96.15%	99.03%	99.39%	99.83%
$\alpha = 0.2$, $\beta = 0.1$	96.06%	98.99%	99.35%	99.85%
$\alpha = 0.2$, $\beta = 0.05$	**96.38%**	**99.12%**	**99.41%**	**99.85%**
$\alpha = 0.15$, $\beta = 0.03$	96.12%	99.05%	99.40%	99.85%
$\alpha = 0.1$, $\beta = 0.03$	96.11%	98.90%	99.37%	99.82%
$\alpha = 0.05$, $\beta = 0.0$	95.98%	99.00%	99.40%	99.84%

**Table 7:** Effectiveness of the different backbones on the CVUSA dataset.

Backbone	CVUSA			
	R@1	R@5	R@10	R@1%
ViT	91.82%	97.76%	98.62%	99.68%
Mamba	89.42%	97.68%	98.84%	99.80%
ResNet34	96.38%	99.12%	99.41%	99.85%

**LRP.** To verify the necessity of LRP, we removed this component, as shown in Table 4. Experimental results show that the model performance exhibits a differentiated trend, the R@1 accuracy drops by 0.56%, while R@10 slightly increases. The data demonstrate the core role of LRP, by balancing multi-scale features, it focuses on enhancing the model’s performance in high-precision matching scenarios, with relatively limited impact on low-stringency metrics. Specifically, the increase in R@10 can be attributed to the fact that after removing LRP, although the feature extraction process loses some local details, it also reduces the model’s risk of overfitting to noise patterns. This allows more candidate samples with similarity to be included in the top 10 matching results, thus showing a positive fluctuation.

**Geo-Feature Encoder.** We conducted an ablation study to demonstrate the effectiveness of the Geo-Feature Encoder. As shown in Table 4, when the Geo-Feature Encoder is removed, all accuracy metrics of the model exhibit a downward trend, with R@1 dropping by 0.66% in particular. This indicates that the module’s hybrid encoding mechanism design can capture both local and global information simultaneously, reflecting its significance for cross-view geolocalization.

**Hybrid Loss.** By comparing the results of the model on the CVUSA dataset under two loss scenarios, as shown in Table 4, it can be observed that the hybrid loss significantly improves the performance of GANet. In addition, we integrated the hybrid loss into the baseline model, as shown in Table 5, and conducted a series of experiments. We found that combining the baseline model with SupCon Loss and Circle Loss, respectively, both led to improved experimental results. Compared with adding a single loss, the combination of SupCon Loss and Circle Loss significantly enhanced the model performance, which indicates that the two can strengthen the learning of hard samples and clarify the convergence target, thereby improving the model performance.

**Geometric Layout Descriptors.** Table 4 also shows the relationship between model performance and the number $K$ of geometric layout descriptors. As $K$ increases, the model achieves better performance. It is noteworthy that there is a significant gap between the results when $K=2$ and $K=8$, which demonstrates the substantial contribution of geometric layout descriptors.

**Hyperparameter Study.** Table 6 presents the retrieval performance of the proposed model on the CVUSA dataset under different combinations of $\alpha$ and $\beta$. The results show that the model performance fluctuates slightly across most parameter settings. It verifies that the method has a certain robustness to weight selection. Specifically, the model achieves the optimal performance when $\alpha =0.2$ and $\beta =0.05$, while either excessively large or small values of $\alpha$ will lead to performance degradation. This indicates that the intensity of semantic constraint needs to be kept within a moderate range. If $\alpha$ is large, the SupCon loss will excessively pull similar sample pairs closer, including some that are actually mismatched. If $\alpha$ is small, the semantic supervision will be insufficient to offset the geometric differences caused by view changes. In contrast, the optimal value of $\beta$ is smaller, and a large $\beta$ will result in significant performance degradation, implying that Circle loss only serves for fine-grained correction in this method and should not dominate the loss function. Thus, $\alpha =0.2$ and $\beta =0.05$ are adopted as experimental configurations.

**Effectiveness of the different backbones.** The results presented in Table 7 demonstrate the performance of three backbone architectures: ViT, Mamba and ResNet34. Among the three backbones evaluated, ResNet34 outperformed the others across all evaluation metrics, achieving the highest accuracy. Specifically, it demonstrated superior results in terms of R@1, R@5 and R@10, indicating its robustness and effectiveness in extracting discriminative features. While ViT also exhibited strong performance, it showed slightly lower accuracy, particularly at the R@1 and R@5 levels. In contrast, Mamba, despite its well-known capabilities, delivered the lowest performance across all metrics, suggesting that its feature extraction abilities may not be as well-suited to the CVUSA dataset. These findings indicate that ResNet34 is the most effective backbone for our approach on the CVUSA dataset, providing a reliable foundation for feature extraction in this task.

###  Visualization analysis

For cross-view geolocalization tasks, learning highly discriminative features such as road orientations and distinctive buildings is crucial. To intuitively analyze the impact of our proposed method on matching performance, we present the feature visualization results of our method and the baseline method in Fig. 5. It can be seen that the Baseline can only learn road information, while the GANet pays more attention to surrounding tree and building features in addition to roads. We attribute the heightened focus of Model on discriminative regions to the efficacy of the Geo-Feature Encoder, which can simultaneously capture global information and local details, providing support for the model to efficiently learn key features.

**Examples of Retrieval.** As an additional qualitative evaluation, this paper also presents the retrieval results of different tasks on the CVUSA and CVACT datasets. We randomly selected three scenarios for each task and computed the top-five results. Correct query results are marked with green boxes, while incorrect images are marked with yellow boxes.

It can be seen from Fig. 6 that GANet performs well in the cross-view geolocalization task. Despite the view difference between ground-level images and aerial images, GANet can still retrieve matching images based on geometric details and global correspondences.

**Attention maps.** We visualize the attention maps of our model on CVUSA as illustrated in Fig. 7. Given a pair of street-view and aerial-view images, we generate the overall attention of each location from the last and first layers in Fig. 7(a), (b), (c), (d). For the street-view query, the first-layer map in Fig. 7(b) shows a relatively scattered pattern, with many dark-blue units and a few medium-response units (cyan and green) scattered across the grid, no single region dominates consistently. In contrast, the last-layer map in Fig. 7(a) becomes significantly clearer. Some units are suppressed to low-level responses in dark blue, while others rise to higher weights (green, yellow, and even brown), thus generating stronger contrast. Shallow-layer attention is scattered and lacks dominant regions. In deep layers, the Encoder reduces the weights of most non-critical positions and focuses attention on a few more crucial and stable discriminative cells. A similar trend is observed for the aerial reference image. In Fig. 7(d), the first-layer attention distribution is relatively uniform and scattered, with most areas showing low-level responses (blue). The last layer highlights a set of strong responses, particularly at the boundaries or isolated positions, the response weights are elevated, changing from blue to cyan, yellow, and orange, while the remaining regions drop to lower values. This indicates that shallow-layer responses to aerial images remain at the coarse texture level, with smooth attention allocation. In deep layers, the Encoder suppresses non-critical backgrounds and enhances discriminative positions.

Comparing the same layer across different views reveals view-dependent differences. In the first layer, the peak positions of the street-view and aerial images are different, and the street-view map exhibits more prominent responses. This aligns with the obvious appearance and differences between the two views. In the last layer, the peak positions are still not completely identical, but both views present a clearer trend: compared with the first layer, more cyan and yellow units appear, and high-response regions become more distinct. This phenomenon can be attributed to the design of the Geo-Feature Encoder, which filters view-specific noise and retains geometry-related cues, thereby stabilizing similarity estimation for cross-view retrieval.

## Conclusion and future works

In our work, we propose a novel and efficient cross-view geolocalization method named GANet. This architecture separates geometric layouts from raw input features to alleviate ambiguities caused by geometric misalignment. Furthermore, we introduce a Hybrid Loss function and experimentally demonstrate that it significantly enhances the performance of the proposed method. Compared with other methods, our approach integrates SSA with convolutional networks and embeds them into feature processing, thereby balancing global information capture and local detail extraction. The performance of the proposed model is significantly superior to that of the baseline methods: in the CVUSA $\rightarrow$ CVACT task, the R@1 metric is improved by 2.79%. In the CVACT $\rightarrow$ CVUSA task, this metric is improved by 3.49%. Extensive experimental results demonstrate that the proposed method exhibits remarkable superiority in cross-view geolocalization tasks. Regarding the limitations of the method proposed in this study including high computational complexity, insufficient speech association capability and limited scenario adaptability. Future research will focus on exploring the design of lightweight models and introducing a language guidance mechanism into cross-view geolocalization tasks.

## Data Availability

The datasets analysed during the current study are available from their original sources. CVUSA is available via the MVRL CVUSA dataset page: https://mvrl.cse.wustl.edu/datasets/cvusa/. CVACT (ACT dataset) is available from the dataset authors upon reasonable request, as described by Liu and Li (2019) and the accompanying project repository.
